# Long-term follow-up of a randomized controlled trial of choline for neurodevelopment in fetal alcohol spectrum disorder: corpus callosum white matter microstructure and neurocognitive outcomes

**DOI:** 10.1186/s11689-022-09470-w

**Published:** 2022-12-16

**Authors:** Blake A. Gimbel, Mary E. Anthony, Abigail M. Ernst, Donovan J. Roediger, Erik de Water, Judith K. Eckerle, Christopher J. Boys, Joshua P. Radke, Bryon A. Mueller, Anita J. Fuglestad, Steven H. Zeisel, Michael K. Georgieff, Jeffrey R. Wozniak

**Affiliations:** 1 University of Minnesota Twin Cities, 2025 E. River Parkway, Minneapolis, MN 55414 USA; 2 Great Lakes Neurobehavioral Center, Edina, USA; 3 Fagron, Inc., Saint Paul, USA; 4 University of North Florida, Jacksonville, USA; 5 University of North Carolina, Chapel Hill, USA

**Keywords:** Fetal alcohol spectrum disorders, Choline, Cognition, Longitudinal studies, Diffusion MRI, Neurite orientation dispersion and density imaging

## Abstract

**Background:**

Fetal alcohol spectrum disorder (FASD) is a lifelong condition. Early interventions targeting core neurocognitive deficits have the potential to confer long-term neurodevelopmental benefits. Time-targeted choline supplementation is one such intervention that has been shown to provide neurodevelopmental benefits that emerge with age during childhood. We present a long-term follow-up study evaluating the neurodevelopmental effects of early choline supplementation in children with FASD approximately 7 years on average after an initial efficacy trial.

**Methods:**

The initial study was a randomized, double-blind, placebo-controlled trial of choline vs. placebo in 2.5 to 5 year olds with FASD. Participants in this long-term follow-up study include 18 children (9 placebo; 9 choline) seen 7 years on average following initial trial completion. The mean age at follow-up was 11.0 years old. Diagnoses were 28% fetal alcohol syndrome (FAS), 28% partial FAS, and 44% alcohol-related neurodevelopmental disorder. The follow-up included measures of executive functioning and an MRI scan.

**Results:**

Children who received choline had better performance on several tasks of lower-order executive function (e.g., processing speed) and showed higher white matter microstructure organization (i.e., greater axon coherence) in the splenium of the corpus callosum compared to the placebo group.

**Conclusions:**

These preliminary findings, although exploratory at this stage, highlight potential long-term benefits of choline as a neurodevelopmental intervention for FASD and suggest that choline may affect white matter development, representing a potential target of choline in this population.

**Trial registration:**

Prior to enrollment, this trial was registered with clinicaltrials.gov (NCT01149538) on June 23, 2010.

## Introduction

Fetal alcohol spectrum disorder (FASD) is an umbrella term describing a group of neurodevelopmental conditions resulting from prenatal alcohol exposure (PAE). The term FASD encompasses subtypes including fetal alcohol syndrome (FAS), partial fetal alcohol syndrome (PFAS), and alcohol-related neurodevelopmental disorder (ARND). Importantly, while these conditions represent a spectrum of severity, research has demonstrated that neurodevelopmental anomalies and cognitive deficits occur across all of these subtypes, including in those without the characteristic facial features associated with PAE (i.e., those with ARND) [1, 2]. These conditions can include neurological impairment, cognitive and behavioral deficits, growth abnormalities, and facial dysmorphology [3, 4]. Global estimates place the prevalence of FASD at 0.8% [5, 6], and recent (and conservative) estimates place the prevalence at 1 to 5% in the USA [5]. Recent evidence indicates that rates of alcohol consumption are increasing globally [7, 8], suggesting a growing risk for alcohol-exposed pregnancies. Despite the high prevalence of FASD, this set of conditions is often unrecognized and misdiagnosed [9, 10], and few interventions have been developed to beneficially alter the lifelong neurodevelopmental course in this population [11, 12]. A large body of evidence has documented a wide range of neurocognitive deficits in FASD, which can include global intellectual deficits, as well as impairment in specific domains of attention, executive functioning, memory, visual-perceptual and motor skills, academic achievement, language skills, adaptive function, and social cognition [3, 13–16]. Even when global cognitive function (i.e., IQ) is not impaired, individuals with FASD can show marked impairment in specific cognitive skills such as executive function and memory [17].

Because of its role in regulating key aspects of brain development [18, 19], including hippocampal development, and its demonstrated capacity to attenuate neurodevelopmental damage from prenatal alcohol exposure in animal models [20–22], the essential nutrient choline has been studied as a potential intervention for the cognitive impairment associated with FASD [23–25]. Choline is known to have direct effects on brain development and function in both typical and atypical development [18]. Choline’s effects are believed to result from its impact on several neurodevelopmental mechanisms and processes. First, choline regulates DNA methylation and gene expression in the brain [26, 27]. It is also integral to the formation of phospholipids involved in cell membranes, axonal growth, and myelination [28, 29]. Choline also acts as a precursor to acetylcholine, which plays an important role in neurotransmission critical for memory and cognition [19, 30–34]. Preclinical studies using animal models of PAE have examined choline supplementation in the perinatal period and demonstrated beneficial effects on brain development [35], motor and behavioral development [36], memory [37, 38], and spatial learning [20]. Our series of studies examining the impact of choline supplementation on brain functions dependent on the hippocampus and on white matter represents a translation of preclinical models to the human. We tested the intervention only up to 5 years of age (when brain growth remains rapid) based on evidence from preclinical studies showing choline’s efficacy during an equivalent window [20]. Similarly, we chose sequential memory measures for our outcome based on preclinical evidence that choline has a direct impact on hippocampal development [39–41].

Few studies have examined choline supplementation in humans (for a comprehensive review, see [24, 27]). Several studies of prenatal (gestational) choline supplementation have suggested that choline supplementation mitigates PAE-related brain volume reductions and improves behavioral outcomes in infants with PAE [25, 42–44]. Research conducted to date suggests there may be a critical period of intervention in the prenatal stage and early childhood (i.e., birth to ages 3–4 years), with prenatal choline supplementation likely leading to larger effects on neurodevelopment in comparison with postnatal supplementation [27]. Our group at the University of Minnesota initially established the safety and tolerability of choline supplementation in children ages 2.5 to 5 years [45]. Our subsequent double-blind, randomized, placebo-controlled trial demonstrated age-related improvements on a developmentally sensitive measure of learning and memory in children ages 2.5 to 5 years who completed 9 months of daily choline compared to those in the placebo group [46]. Notably, we observed a steeper improvement in memory performance in younger children (ages 2.5 to 4 years) compared to older children (ages 4 to 5 years) in this sample. We conducted a 4-year follow-up study of children with PAE who participated in our initial clinical trial [23]. Larger and more consistent benefits for choline were observed at this later point in development. Participants in the choline group demonstrated higher nonverbal intelligence, visual-spatial skill, working memory ability, and verbal memory performance, as well as fewer parent-rated behavioral symptoms of ADHD compared to the placebo group. In contrast to these studies, a double-blind, placebo-controlled trial conducted by Nguyen and colleagues [47] found no group differences following a much shorter (6 weeks) choline supplementation in older children with PAE ages 5 to 10 years. Together, this research suggests there may be a critical period in early development for choline supplementation, with a protracted course of cognitive benefits that emerge over time. To our knowledge, potential long-term effects of early choline supplementation beyond 4 years have not been investigated, highlighting the need for further longitudinal research such as the study reported here.

In considering potential neurodevelopmental outcomes from choline supplementation, it is worth noting that the literature contains several decades of neuroimaging work highlighting a diverse range of neurological abnormalities associated with PAE, including reduced overall brain volume and regional gray matter volumes, differences in cortical thickness and gyrification, and abnormal gray and white matter longitudinal growth trajectories [48–50]. White matter (WM) abnormalities are among the most well-replicated neuroimaging findings in PAE and are thought to contribute to impaired functional connectivity and prominent deficits in attention and executive function in individuals with PAE [51–55]. Diffusion abnormalities in the corpus callosum (CC) are frequently observed in individuals with PAE and have been linked with other clinical features such as facial dysmorphology [56–58]. In typical development, the CC forms early in gestation and undergoes extensive morphological and volumetric change throughout childhood, with a period of rapid development during late childhood and adolescence [59, 60]. Frequent findings in PAE include reduced fractional anisotropy (FA), higher mean diffusivity (MD), and higher radial diffusivity (RD) compared to typically developing controls [54]. A number of studies have also observed shape abnormalities and overall volume reductions in the CC, particularly in posterior regions such as the splenium [54, 61, 62]. Such abnormalities in CC volume and microstructure have been associated with neurocognitive performance on tasks of eyeblink conditioning [63], working memory [55], mathematics [64], language and reading skill [65], and interhemispheric information transfer [66].

To date, a majority of studies examining white matter abnormalities in PAE have used traditional diffusion tensor imaging metrics such as FA and MD. Newer diffusion modeling techniques have the potential to provide greater specificity regarding WM microstructural abnormalities associated with neurodevelopmental conditions like FASD [54, 67]. For example, neurite orientation dispersion and density imaging (NODDI) [68] is a biophysical and multicomponent model that uses diffusion MRI data to characterize the density of neurites (i.e., axons and dendrites) as well as the alignment (dispersion) of tissue fibers within each voxel [69, 70]. The neurite density index (NDI) represents the intracellular volume fraction and is thought to reflect the density of axons (especially in white matter) and potentially dendrites (especially in gray matter). Neurite density is known to be functionally relevant, as evidenced by well-established relationships between dendritic arbor complexity and cognitive function [71]. In white matter, greater NDI values suggest axonal growth, greater axonal density, and/or myelination [70]. The orientation dispersion index (ODI) represents the angular variation of diffusion orientation and is thought to reflect the degree of bending and fanning of axons in white matter [68]. To our knowledge, no research has been conducted to date using NODDI to characterize WM abnormalities in PAE. NODDI has been used in several studies of neurodevelopmental conditions associated with WM microstructural abnormalities, such as prematurity [69, 72]. Given the role of choline in lipid synthesis, myelination, and axonal growth, as well as the greater specificity provided by NODDI metrics regarding tissue microstructure, this novel approach is well-suited for exploring potential microstructural WM differences associated with early choline supplementation in children and adolescents with PAE.

Here, we present data from a long-term (i.e., 7 years on average since initial trial) follow-up study of child participants treated with either choline or placebo in our initial randomized controlled trial of choline supplementation at ages 2.5 to 5 years of age. Examination of diffusion MRI and cognitive testing data from this long-term follow-up evaluation allowed us to explore potential lasting benefits of an early choline supplementation intervention targeting neurodevelopment. We examined domain-specific outcomes focusing on attention and executive functioning. Diffusion-weighted MRI and the NODDI biophysical model were used to explore potential differences in CC WM microstructure between treatment groups. Our reason for focusing on NODDI rather than additionally examining traditional diffusion metrics (e.g., FA, MD) was twofold: first, due to the small sample size, we aimed to limit the number of outcome measures examined; second, literature to date suggests NODDI provides a more biologically relevant method of modeling diffusion data due to its multi-compartment approach and better correlation with histology compared to traditional diffusion tensor modeling [69]. Therefore, in this study, we chose to implement the most advanced, most biologically relevant, and (theoretically) most sensitive diffusion imaging model. In this study, we specifically focused on the CC because of the large body of evidence highlighting the vulnerability of this region to PAE [54], as well as associations of CC diffusion anomalies to neurocognitive functioning in PAE [55, 63–66]. We also explored the relationship of CC microstructure to facial dysmorphology given previous findings in some studies of a relationship between CC anomalies (e.g., shape, thickness, diffusion) and facial features in PAE [56–58, 73, 74]. Given the limited sample size of this study, analyses were exploratory in nature.

## Materials and methods

### Parent-study methods and participants

Participants in the current long-term outcome study were children with PAE who took part in an earlier clinical trial of choline supplementation [46]. Detailed materials and methods for this earlier clinical trial and the 4-year follow-up study have been previously published [23, 45, 46] and will, therefore, be briefly summarized here. This was a randomized, double-blind, placebo-controlled study in which participants were randomized to receive either choline (1.25 g choline bitartrate powder mix delivering 513 mg choline) or matching placebo for 9 months (NCT01149538). A complete description of methods and procedures used in this clinical trial was reported in [45], and results of the initial trial were reported in [46].

**Fig. 1:**
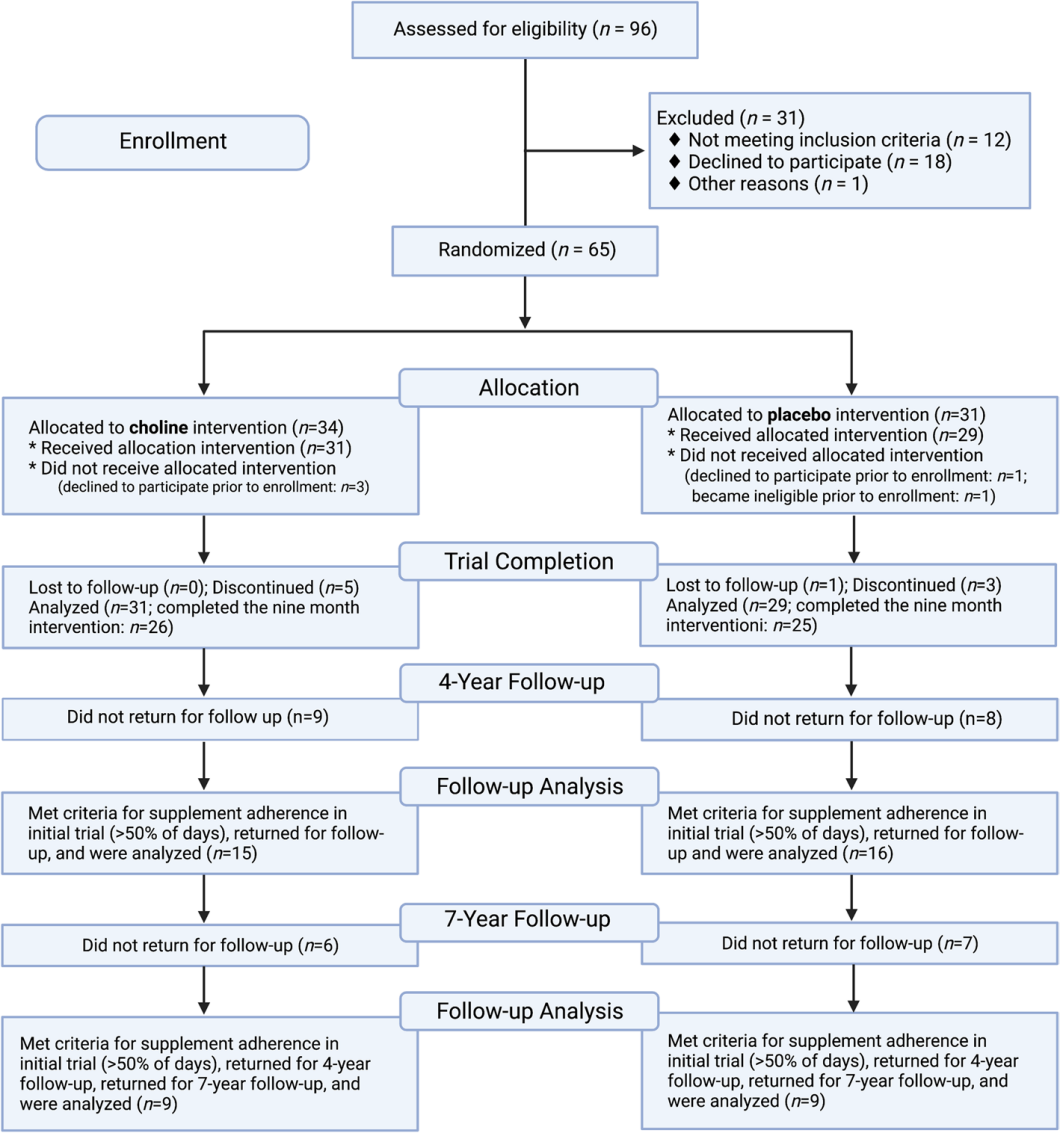
CONSORT flow diagram for initial randomized clinical trials, 4-year follow-up study, and 7-year follow-up study

A total of 60 participants with FASD between the ages of 2.5 to 5 years were enrolled between June 2010 and May 2014 following a screening process to determine eligibility as well as IRB-approved consent process (Fig. 1). Initial exclusion criteria were the presence of another developmental disorder (e.g., autism, Down syndrome), low birth weight (< 1500 g), neurological disorder, traumatic brain injury, or other medical conditions affecting the brain. Participants were primarily recruited from the University of Minnesota Fetal Alcohol Spectrum Disorder Clinic and/or Adoption Medicine Clinic. Participants received the allocated intervention of choline or placebo (1:1 allocation to parallel groups), of which 85% (*n* = 51) completed the 9-month trial [46]. Investigators, staff, and participants were blinded to the treatment assignment. Parents administered the study drug to participants once daily for 9 months. Compliance was measured by calendar logs and packet counts with both groups averaging 88% days with a dose taken. In the initial follow-up, the Mullen Scales of Early Learning [75], which served as a primary outcome and a measure of global cognitive functioning, was administered at baseline and 9 months (study completion). An elicited imitation (EI) paradigm [76, 77] provided a measure of hippocampal-dependent sequential memory at baseline and 9 months. The Child Behavioral Checklist (CBCL) [78] was completed by parents at baseline and study completion.

### Follow-up methods

Of those who completed the initial trial, 31 participants returned for a 4-year follow-up visit (15 choline and 16 placebo) between December 2015 and November 2017 to evaluate the longer-term neurodevelopmental effects of choline supplementation [23] (Fig. 1). New clinical diagnoses were recorded at the time of the 4-year follow-up visit. In the choline group, new diagnoses included 1 child with dyslexia and attention-deficit/hyperactivity disorder (ADHD). In the placebo group, new diagnoses included 1 child with autism spectrum disorder, 1 child with other specified depressive disorder and unspecified ADHD, and 1 child with anxiety. Because of the small number of diagnoses, we did not conduct any additional analyses on these data. A total of 17 participants (9 choline and 8 placebo) from the initial trial did not return for 4-year follow-up (lost contact or declined participation). Of the participants who returned, 4 (13%) were characterized as having FAS, 13 (42%) as having PFAS, and 14 (45%) as having alcohol-related neurodevelopmental disorder (ARND) based on the modified Institute of Medicine (IOM) criteria [79] with the height, weight, and head circumference measurements taken at initial enrollment [46]. Participants completed several cognitive measures assessing intellectual function (IQ), memory, attention, executive functioning, and inhibitory control. For more details about the 4-year follow-up, please refer to [23].

Subsequently, participants who were part of the 4-year follow-up study were asked to return for this long-term follow-up visit. The mean long-term follow-up duration was 7 years (range 4–10 years) after initial trial completion. A total of 18 participants (9 choline and 9 placebo; 5 females in each group) returned for neuroimaging and additional cognitive testing (Fig. 1). Participants were ages 8 to 15 years at long-term follow-up (mean age 11.0). A total of 6 choline and 7 placebo group participants did not return for this visit (lost contact, declined participation, or moved). Of the participants who returned, 5 (28%) had a diagnosis of FAS, 5 (28%) had a diagnosis of PFAS, and 8 (44%) had a diagnosis of ARND. Furthermore, within the choline group, 2 (22%) had FAS, 2 (22%) had pFAS, and 5 (57%) had ARND. In the placebo group, 3 (33%) had FAS, 3 had pFAS (33%), and 3 (33%) had ARND. All subtypes require some form of cognitive or behavioral impairment and a history of PAE. FAS also requires facial dysmorphology, growth deficiency, and a brain growth deficiency. In addition to cognitive or behavioral impairment, PFAS requires facial dysmorphology. ARND only requires a history of PAE and cognitive or behavioral impairment [4]. Research staff contacted participants via phone, email, and mailed letters. Participants were re-consented using IRB-approved consent and assent processes and forms. During a single visit, participants completed neurocognitive testing and MRI scanning. The last of these visits took place in 2021.

Given attrition of participants at the 4-year and 7-year follow-up studies, we compared participants who did and did not return in terms of overall cognitive function using independent-samples *t*-tests to test for potential selection bias in the returning participants. Participants who returned for the 4-year and 7-year follow-up studies did not differ from those who did not return regarding baseline cognitive function measured at the start of the original choline supplementation trial. Similarly, participants who did not return for the 7-year study did not differ in terms of cognitive function measured during the 4-year study.

### Executive functioning

Participants completed the Digit Span and Picture Span subtests of the Wechsler Intelligence Scale for Children, 5th Edition (WISC-IV) [80] and the Trail-Making and Color-Word Interference subtests from the Delis-Kaplan Executive Functioning System (D-KEFS) [81]. These cognitive tests measure higher-level executive functioning skills (i.e., working memory, inhibitory control, switching/cognitive flexibility) as well as “lower-order” processes (e.g., visual scanning, attention, processing speed). Test scaled scores (i.e., mean of 10, standard deviation of 3) were used in all analyses.

### MRI acquisition and processing

MRI data were acquired on a Siemens 3T Prisma scanner (Erlangen, Germany) equipped with a 32 channel head coil. For each participant, T1-weighted and T2-weighted scans were acquired using custom pulse sequences, which included automatic real-time motion detection and k-space line rejection and replacement software [82]. Pulse sequence parameters were chosen to match those used in the Lifespan Human Connectome Project in Development [83]. Selected parameters are described in Table 1.

**Table 1:** Diffusion MRI acquisition parameters

Sequence	Imaging parameters
T1 weighted	Multi-echo MP-RAGE sequence with *TR* = 2500 ms, *TE* = 1.8/3.6/5.4/7.2 ms, *TI* = 1000 ms, voxel size = 0.8 mm isotropic, flip angle = 8°
T2 weighted	SPACE sequence with *TR* = 3200 ms, *TE* = 564 ms, voxel size = 0.8 mm isotropic, variable flip angle
Diffusion weighted	Multiband EPI sequence with *TR* = 3230 ms, *TE* = 89.2 ms, voxel size = 1.5 mm isotropic, *MB* = 4. Four consecutive runs of about 5:31 each were acquired with 2 shells (*b* = 1500 and 3000 s/mm^2^) and 46 directions each and 7 volumes with *b* = 0 s/mm2.^a^

^a^Four participants had only 2 diffusion-weighted scans, and in two participants, one of the DWI scans was rerun; all data were included in this analysis

*TR* Repetition time, *TE* Echo time, *MB* Multiband, *ms* Milliseconds

All MRI data were initially preprocessed using the Human Connectome Project’s Minimal Preprocessing Pipeline (v4.0.1) [84]. For structural data, these steps included alignment of the T1-weighted volume to the T2-weighted volume and correction for gradient distortion and intensity bias before running FreeSurfer (v6.0.0), which performs subcortical segmentation, creates pial and white matter surface meshes, and neocortical parcellation [85, 86]. Diffusion preprocessing included rigid AC-PC alignment to the structural images, correction for susceptibility-related distortions using FSL’s top-up, and eddy current distortion correction and slice outlier replacement using FSL’s eddy tool (v 6.0.1).

**Fig. 2:**
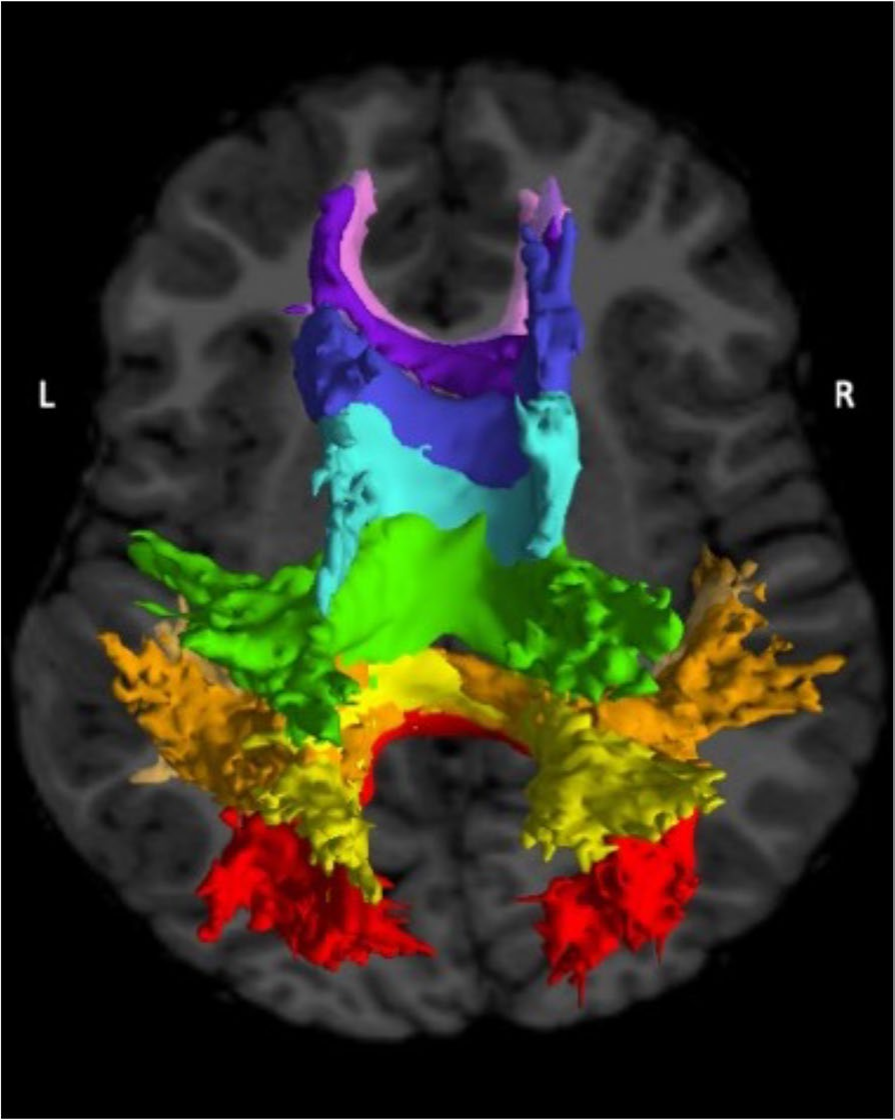
Axial view of eight corpus callosum tracts generated by tractography (anterior/top of figure to posterior/bottom of figure). Pink, rostrum; purple, genu; dark blue, body (prefrontal); light blue, body (premotor); green, body (central); yellow, body (parietal); orange, body (temporal); red, splenium

Tractography to delineate major white matter tracts was carried out using the TRActs Constrained by Underlying Anatomy (TRACULA) tool available in the FreeSurfer v7.2.0 release [87]. TRACULA identifies 42 major white matter tracts based on probabilistic tractography informed by expertly defined anatomical priors. Voxel-wise NODDI metrics were estimated using the Accelerated Microstructure Imaging via Convex Optimization (AMICO) toolkit [88]. Finally, for each metric and tract of interest, tract-wise averages were computed, weighting each voxel based on likelihood of tract membership and excluding any voxels with less than 20% of the robust maximum probability of tract membership. For this analysis, we focused on the eight major callosal tracts defined by TRACULA (Fig. 2): the central, parietal, premotor, prefrontal, and temporal projections from the callosal body and the genu, rostrum, and splenium. An experienced rater (DJR) inspected all raw images, FreeSurfer segmentations and surface parcellations, tractography results, and NODDI scalar maps. The rater determined that no data should be excluded based on quality or aberrant processing.

### Statistical analyses

Statistical analyses were performed with R version 4.1.1 [89]. First, independent sample *t*-tests and chi-square tests were used to examine treatment group differences (choline vs. placebo) in demographic variables and whole brain/hemispheric volume at follow-up visit. Initial data exploration (e.g., visual inspection of histograms) and Shapiro-Wilk tests revealed several outcome variables to be non-normally distributed (i.e., corpus callosum ODI values in the genu, premotor, body [central], body [parietal], and body [temporal]; D-KEFS Trail-Making Test visual scanning, number sequencing, letter sequencing, motor speed; D-KEFS Color-Word Interference Test color naming). As such, Mann-Whitney *U*-tests were used to compare treatment groups on mean NODDI metrics (NDI and ODI) in defined parts of the corpus callosum and cognitive outcomes at follow-up visit. Cohen’s *d* effect sizes were calculated and interpreted as small (*d* = 0.2), moderate (*d* = 0.5), or large (*d* = 0.8; [90]). Given known microstructural abnormalities of the corpus callosum associated with PAE, Kendall’s tau correlations were used to determine the relationship between NODDI metrics (in tracts with significant group differences in microstructure) and executive function test performance at follow-up testing (conducted at the same time as the follow-up MRI scan) at the whole-group level. Significance values were reported with and without correction for multiple comparisons given the number of correlations performed (11 in total) and the exploratory nature of these analyses. Significance values were corrected for multiple comparisons using the Holm-Bonferroni procedure [91, 92]. Lastly, exploratory analyses were performed to examine the relationship of facial dysmorphology to corpus callosum microstructure using both Kendall’s tau correlations (with number of dysmorphic facial features as a continuous variable [0 to 3 features]) and Mann-Whitney *U*-tests (with facial dysmorphology as a dichotomous variable [dysmorphic face = 2 or more facial features]).

## Results

### Participant characteristics

As shown in Table 2, the choline and placebo groups did not differ significantly on demographic variables including age at enrollment and age at follow-up evaluation, sex, ethnicity, and handedness (there were two participants for whom handedness data were not available). The groups also did not differ significantly with regard to total intracranial volume, overall intelligence, or physical characteristics (i.e., growth deficiency, microcephaly, and dysmorphic facial features). Similar to other studies of children with PAE, mean intelligence at 4-year follow-up (as measured by the Stanford-Binet Intelligence Scales, Fifth Edition; [93]) was significantly lower than the mean of the test normative sample (i.e., a mean IQ of 100). That is, the choline group mean IQ at 4-year follow-up (standard score of 90.5) was significantly lower than the test normative sample, *t*(7) = −2.63, *p* = 0.01, as was the placebo group mean IQ at 4-year follow-up (83.56), *t*(8) = −2.28, *p* = 0.02.

**Table 2:** Demographic characteristics of participants included in analyses

*N*(%) or mean (SD)	Choline (*n* = 9)	Placebo (*n* = 9 )	Statistical test
*Age at enrollment (years)*^*a*^	3.54 (0.91)	4.18 (0.66)	*t*(15) = −1.70, *p* = 0.11
*Age at follow-up evaluation (years)*	10.56 (1.81)	11.44 (1.67)	*t*(15) = −1.08, *p* = 0.29
*Total intracranial volume cm3*^*b*^	1461.9 (119.6)	1437.7 (74.9)	*t*(13) = 0.51, *p* = 0.61
*Intelligence quotient*^*c*^	90.5 (10.2)	83.56 (21.63)	*t*(12) = 0.86, *p* = 0.41
*Sex (female)*	5 (55.6%)	5 (55.6%)	*χ*^2^ (1) = 0, *p* = 1.00
*Ethnicity (Hispanic)*	1 (11.1%)	1 (11.1%)	*χ*^2^ (2) = 1.07, *p* = 0.59
*Race*^*d*^			
*American Indian/Alaska Native*	0	3 (33.3%)	*χ*^2^ (1) = 5.63, *p* = 0.02
*Asian*	1 (11.1%)	1 (11.1%)	*χ*^2^ (1) = 0.89, *p* = 0.35
*Black or African American*	0	2 (22.2%)	*χ*^2^ (1) = 4.44, *p* = 0.04
*White*	5 (56.6%)	1 (11.1%)	*χ*^2^ (1) = 4, *p* = 0.04
*Multiracial*	2 (22.2%)	2 (22.2%)	*χ*^2^ (1) = 1.27, *p* = 0.26
*Unknown*	1 (11.1%)	0	*χ*^2^ (1) = 0.19, *p* = 0.66
*Handedness (right)*^*e*^	8 (88.9%)	7 (77.8%)	*χ*^2^ (1) = 2.01, *p* = 0.16
*IOM diagnostic category*			*χ*^2^ (2) = 0.90, *p* = 0.63
*FAS*	2 (22.2%)	3 (33.3%)	
*Partial FAS*	2 (22.2%)	3 (33.3%)	
*ARND*	5 (56.6%)	3 (33.3%)	
*Physical characteristics*			
^*f*^*Growth deficiency*	3 (33.3%)	4 (44.4%)	*χ*^2^ (1) = 0.23, *p* = 0.63
^*g*^*Microcephaly*	4 (44.4%)	5 (56.6%)	*χ*^2^ (1) = 0.22, *p* = 0.64
^*h*^*Dysmorphic face*	4 (44.4%)	6 (66.7%)	*χ*^2^ (1) = 0.90, *p* = 0.34

Demographics are from the 18 participants included in the analyses. Age at follow-up evaluation ranged from 8 to 15 years. Participants were excluded from this analysis if significant data were missing. Of the initial eligible pool of 20 participants who returned for follow-up, two participants who did not complete neuroimaging were eliminated

^a^Age at enrollment = age at initial clinical trial enrollment (participants age 2.5–5)

^b^Total intracranial volume = total intracranial volume at time point at follow-up evaluation

^c^Intelligence quotient was measured at the 4-year follow-up visit (full-scale IQ standard score)

^d^Chi-square tests reflect comparisons of each racial group to the proportion of participants who identified as White (e.g., proportion of participants who identified as multiracial to proportion of those who identified as White)

^e^Missing handedness data for two participants

^f^Height or weight ≤ 10%ile

^g^Head circumference ≤ 10%ile

^h^At least two of the following: palpebral fissure length ≤ 10%ile, thin vermillion border, smooth philtrum (4 or 5 on lipometer scale)

Despite randomization, there were significant group differences in racial identity between the choline and placebo groups. In general, participants in the placebo group who returned for follow-up evaluation were more racially diverse than those in the choline group. The three racial comparisons that were statistically significant included the proportion of American Indian/Alaska Native to White individuals (*χ*^2^ (1) = 5.63, *p* = 0.02), Black/African American to White (*χ*^2^ (1) = 4.44, *p* = 0.04), and White to non-White (*χ*^2^ (1) = 4.00, *p* = 0.04).

### Executive functioning

Participants in the choline group demonstrated better mean performance than those in the placebo group across the majority of executive function measures at follow-up testing (Table 3). There were no significant group differences for the WISC-V working memory subtests (Digit Span and Picture Span) or the D-KEFS Color-Word Interference Test. Participants in the choline group demonstrated significantly higher mean performance than those in the placebo group on the D-KEFS Trail Making Trial of motor speed (Cohen’s *d* = 1.14; 18.2% difference) measuring the speed of visual-motor processing, representing a large effect size. For the D-KEFS Trail Making Color-Word Interference Test color naming trial, there was a trend (*p* = 0.05) toward higher performance in the choline group compared to the placebo group, representing a large effect size (*d* = 1. 27) and a 63.2% difference. Although the Color-Word Interference Test trials of inhibition and inhibition/switching were not statistically significant, the choline group demonstrated numerically higher performance than the placebo group. Effect sizes were moderate for group differences in inhibition (*d* = 0.72; 41.6% difference) and inhibition/switching (*d* = 0.78; 34.1% difference) performance.

**Table 3:** Treatment group differences in executive function performance at follow-up evaluation

Median; mean (SD) {*n*}	Placebo	Choline	Statistic	Significance	Effect size
WISC-V					
Digit Span	6.0; 6.88 (3.68) {8}	9.0; 8.29 (2.43) {7}	*W* = 35.5	0.41	0.45
Picture Span	10.0; 9.29 (2.63) {7}	11.0; 10.14 (4.38) {7}	*W* = 31.5	0.40	0.23
D-KEFS Trail Making Test					
Visual scanning	9.5; 9.12 (3.60) {8}	10.0; 9.29 (3.90) {7}	*W* = 32.0	0.68	0.04
Number sequencing	11.0; 9.88 (3.98) {8}	12.0; 11.57 (0.98) {7}	*W* = 36.5	0.34	0.58
Letter sequencing	9.5; 8.50 (5.10) {8}	10.0; 8.0 (4.90) {7}	*W* = 27.5	1.00	−0.09
Number/letter sequencing	10.5; 7.0 (3.59) {8}	11.0; 9.14 (3.14) {7}	*W* = 1.2	0.91	0.61
Motor speed	10.0; 9.12 (3.18) {8}	12.0; 11.86 (1.21) {7}	*W* = 45.5	0.04*	1.14
D-KEFS Color-Word Interference Test					
Color naming	3.0; 4.38 (2.92) {8}	10.0; 8.43 (3.41) {7}	*W* = 45.0	0.05	1.27
Word reading	8.0; 7.75 (2.05) {8}	9.0; 9.86 (1.68) {7}	*W* = 42.0	0.11	1.13
Inhibition	3.5; 5.62 (5.01) {8}	10.0; 8.57 (2.76) {7}	*W* = 37.5	0.29	0.72
Inhibition/switching	7.5; 6.88 (4.67) {8}	10.0; 9.71 (2.06) {7}	*W* = 41.0	0.14	0.78

*WISC-V* Wechsler Intelligence Scale for Children, 5th Edition, *D-KEFS* Delis-Kaplan Executive Function System

*Significance *p* < .05

### White matter microstructure

Mann-Whitney *U*-tests were used to examine treatment group differences (placebo vs choline) for NODDI measures in the corpus callosum (Table 4). The choline group demonstrated significantly lower ODI (more coherent fibers with less bending/fanning) in the splenium of the corpus callosum compared to the placebo group, which represented a large effect (*d* = −1.26; 20.0% difference). No other significant treatment group differences were found in corpus callosum ODI. Although not statistically significant, the choline group showed marginally lower ODI in the temporal region of the body of the corpus callosum compared to placebo (6.9% difference), representing a moderate effect (*d* = −0.63). For NDI, there were no treatment group differences in the corpus callosum.

**Table 4:** Treatment group differences in corpus callosum NODDI ODI at follow-up evaluation

Median; mean (SD) {*n*}	Placebo	Choline	Statistic	Significance	Effect size
Rostrum	0.11; 0.11 (0.02) {9}	0.10; 0.11 (0.02) {9}	*W* = 29.0	0.34	0
Genu	0.13; 0.13 (0.03) {9}	0.13; 0.12 (0.02) {9}	*W* = 33.0	0.55	−0.39
Body prefrontal	0.13; 0.13 (0.02) {9}	0.14; 0.13 (0.02) {9}	*W* = 46.0	0.67	0
Body premotor	0.11; 0.12 (0.03) {9}	0.12; 0.12 (0.04) {9}	*W* = 34.0	0.61	0
Body central	0.12; 0.12 (0.02) {9}	0.10; 0.11 (0.01) {9}	*W* = 21.0	0.09	−0.13
Body parietal	0.13; 0.14 (0.03) {9}	0.12; 0.12 (0.01) {9}	*W* = 18.0	0.05	−0.21
Body temporal	0.14; 0.15 (0.02) {9}	0.14; 0.14 (0.01) {9}	*W* = 30.0	0.39	−0.63
Splenium	0.11; 0.11 (0.01) {9}	0.09; 0.09 (0.02) {9}	*W* = 15.0	0.02*	−1.26

*CSF* Cerebrospinal fluid

*Significance *p* < .05

### Relationship of cognitive performance to white matter microstructure

**Fig. 3:**
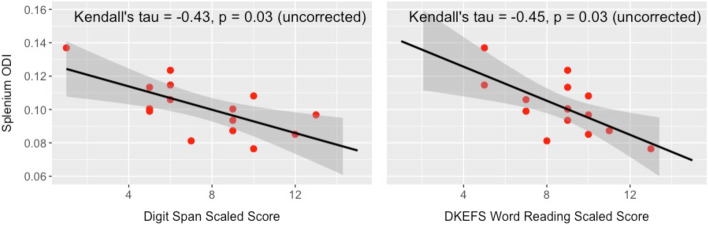
Scatterplots illustrating significant whole-group correlations (not corrected for multiple comparisons) between corpus callosum splenium ODI and executive function performance at follow-up evaluation. Lower ODI values are associated with better performance

Correlation analyses were performed with the whole sample (i.e., collapsing diagnostic groups) to explore the relation of cognitive performance to white matter microstructure in the splenium of the corpus callosum because this region showed a significant difference between choline and placebo groups. Correlations with Kendall’s tau indicated performance on two executive function measures were negatively correlated with splenium ODI (Fig. 3): Digit Span scaled score (*τ*_b_ = −0.43, *p* = 0.03) and D-KEFS Word Reading scaled score (*τ*_b_ = −0.45, *p* = 0.03). Correction of significance values for multiple comparisons using the Bonferroni-Holm procedure revealed that neither correlation survived correction, possibly due to the small sample size and limited statistical power. Importantly, higher scores on these executive function measures indicate better performance, whereas lower ODI values represent less bending and fanning of axons (i.e., more coherent fibers). As such, the negative correlations observed here are in the expected direction: better executive function performance was associated with lower ODI values in the splenium. No other significant correlations were found.

### Relationship of facial dysmorphology to corpus callosum white matter microstructure

**Fig. 4:**
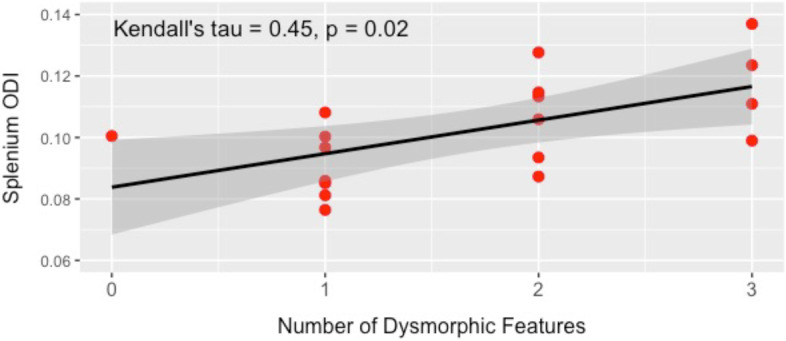
Scatterplot illustrating a significant whole-group correlation between corpus callosum splenium ODI (imaged at follow-up evaluation) and number of dysmorphic facial features identified as baseline evaluation. Lower ODI values are associated with fewer dysmorphic features

The relationship between ODI in the splenium of the corpus callosum and facial dysmorphology (evaluated at baseline assessment prior to commencing choline supplementation) was also examined (Fig. 4). Results of a Kendall’s tau correlation between the total number of dysmorphic facial features (i.e., 0 to 3) and splenium ODI revealed a significant positive correlation in the whole sample (*τ*_b_ = 0.45, *p* = 0.02); a higher number of dysmorphic facial features was associated with lower microstructural organization. Similarly, a Mann-Whitney *U*-test with facial dysmorphology used as a dichotomous variable revealed significant differences between groups in splenium ODI. Participants without a clinically dysmorphic face (i.e., fewer than two facial features; median = 0.09) showed lower splenium ODI (better organization) compared to those with a clinically dysmorphic face (i.e., two or more facial features; median = 0.11, *W* = 12.0, *p* = 0.01).

## Discussion

Data presented here from our longitudinal RCT follow-up study of children with FASD suggest potential long-lasting benefits to executive function skills approximately 7 years on average following early choline supplementation. These 7-year outcome data are consistent with our 4-year outcome data, which demonstrated benefits in nonverbal IQ, visual-spatial skills, working memory, verbal memory, and ADHD-related behavioral problems [23], and these effects extend to an age where the changes likely reflect stable long-term advantages. In a continuing trend first reported at the 4-year follow-up, the 7-year effect sizes were also larger and more consistent across measures than those initially observed immediately following trial completion [46]. Notably, effect sizes for several measures of executive function were moderate to large in magnitude in comparison with the moderate effect sizes (and nonsignificant group differences) observed at the 4-year point on similar tests of executive function [23]. Early preclinical investigations suggested a regionally specific effect of choline on hippocampal structure and function [94, 95], which was further supported by results from our first clinical trial in young children demonstrating an effect for postnatal choline supplementation on a hippocampal-dependent memory task but not overall cognitive function [46]. Indeed, similar studies investigating nutritional supplementation such as long-chain polyunsaturated fatty acid early in life suggest a “scaffolding” effect in which effects of intervention on early-developing foundational processes support later development of brain structures and functions (e.g., IQ, executive function) [96]. As such, one would expect long-term effects to be in a wider domain than the original effects of an early intervention [97], which aligns with findings of the current study of wider gaps in executive function performance at 7-year follow-up between choline and placebo participants compared to 4-year follow-up. Such treatment group differences may continue to widen with age, highlighting the need for further longitudinal research with longer-term follow-up evaluations.

In this 7-year follow-up sample, participants in the choline group had higher mean scores across a majority of executive function tests compared to those in the placebo group. A significant group differences (choline > placebo) was observed on the motor speed trial of the D-KEFS Trail Making Test, and a trend-level group difference (choline > placebo) was observed on the color naming trial of the D-KEFS Color-Word Interference Test. These results highlight the continued neurodevelopmental benefits of early choline supplementation in children with FASD. Our findings also suggest that benefits to aspects of executive functioning performance from early supplementation may become increasingly apparent in middle childhood and adolescence, which aligns with previous work suggesting there may be an early developmental window for choline’s therapeutic effects, and that such effects may emerge and increase across time [24, 47]. Future research should continue to investigate the neurocognitive benefits of early choline supplementation, including variations in dose, duration, and timing of choline in early childhood, as well as potential neurocognitive and behavioral benefits continuing into late adolescence and adulthood. An ongoing study by our group at the University of Minnesota (NCT05108974) is investigating several of these important questions, including evaluation of a weight-adjusted dose and random assignment of children with PAE ages 2.5–5 years to receive either 3 months of daily choline plus 6 months of placebo or 6 months of daily choline plus 3 months of placebo.

Our finding of a choline benefit for measurable aspects of executive function (i.e., processing speed) at 7 years post-intervention is encouraging and could plausibly translate to real functional benefits for children from early choline supplementation. Executive function skills develop along a nonlinear trajectory during childhood and adolescence [98–100] and parallel the maturation of the cerebral cortex and white matter [101–103]. In addition, executive function in childhood and adolescence is associated with long-term outcomes across the lifespan including social-emotional functioning, academic achievement, and risk for psychopathology [104]. Impairments in attention and executive function are hallmark features of FASD and have been repeatedly demonstrated in several studies [52, 105–107]. As such, these are meaningful targets for intervention in FASD, and our findings suggest that early choline supplementation may play an important role in conferring neurodevelopmental benefits for this uniquely vulnerable skillset.

Performance advantages for the choline group on the color-naming trial of the D-KEFS Color-Word Interference Test and the motor speed trial of the D-KEFS Trail Making Test may be consistent with the potential underlying white matter change observed here following choline supplementation. These tasks may reflect “lower-order” processes (e.g., visual scanning, attention to the task, speed of information processing, visuo-motor speed) that are necessary but not sufficient for performance on higher-level EF skills, such as inhibitory control and shifting/cognitive flexibility [108]. Age-corrected scaled scores are generated from the task completion time in seconds, and as such, these tasks provide a metric for lower-order skills such as speed of visuomotor processing and verbal output [109]. Consistent with our findings, past studies have documented an association between microstructural abnormalities in the posterior CC and cognitive performance on tasks measuring working memory, visual-motor integration, memory, and IQ [55, 110, 111]. Future studies with more than one neuroimaging time point could further examine relationships between choline supplementation and cognitive outcome via underlying brain mechanisms including white matter microstructure.

Unique to this study is the examination of diffusion MRI data using a biophysical model (i.e., NODDI) that allows for greater specificity with regard to white matter microstructure compared to traditional diffusion tensor imaging metrics such as FA and MD [54, 67]. We demonstrate white matter microstructural differences between treatment groups, suggesting early choline supplementation may affect white matter development. Specifically, we found significant group differences in white matter microstructure in the splenium of the corpus callosum (i.e., lower ODI). We also observed that, collapsing across groups, higher ODI values were associated with a greater number of dysmorphic features, consistent with several previous studies showing an association between corpus callosum integrity (volume and microstructure) and facial dysmorphology in PAE samples [56–58]. Abnormalities in the corpus callosum (genu, body, isthmus, and splenium) have been repeatedly demonstrated in PAE samples using traditional diffusion MRI metrics [54]. Specific findings have included reductions in FA and/or increases in MD, RD, or AD in these regions of the corpus callosum [55, 61, 65, 110, 112]. Here, we show that abnormalities in the corpus callosum (specifically the splenium) may be partially ameliorated by early choline supplementation. It is noteworthy that we did not find differences in NDI given the role of choline in lipid synthesis and myelination. Age-related increases in NDI in WM are thought to reflect myelination, axonal growth, or increases in axon density, and WM NDI tends to align better with FA than ODI [70]. In contrast, ODI changes little across child and adolescent development and provides a measure of neurite dispersion that points to coherence of axons and geometry [70]. Our findings of lower ODI in the splenium in the choline group could possibly reflect a beneficial developmental effect of early choline supplementation in terms of the structure and organization of axons in the corpus callosum. However, given that NODDI has been used in a limited number of studies with pediatric samples [69] and to our knowledge has not yet been used in studies of individuals with PAE, interpretation of our results remains provisional at this point. Together, our findings of treatment group differences in both brain microstructure and related cognitive function (e.g., processing speed) suggest a biologically plausible long-term effect of early choline supplementation in PAE.

Several limitations should be considered in interpreting results of the current study and informing future investigations. First, as a result of attrition, a limited number of initial choline supplementation trial participants returned for follow-up evaluation. Nonetheless, the returning groups were well matched with each other. Importantly, despite randomization, there were differences in racial identity between treatment groups. The size of our sample and limitations in statistical power meant that we were unable to explore potential confounding effects of group differences in racial identity on neuroimaging and neurocognitive outcomes, highlighting an important consideration for future research. Although the small sample size available for analysis at long-term follow-up limits the generalizability of our findings, it is noteworthy that significant treatment group differences and biologically plausible neuroimaging results were nevertheless identified in this small sample. A second limitation is the range of duration (ranging from 4 to 10 years) between trial completion and long-term evaluation in our sample. This is relevant because brain development and cognitive performance changes occur rapidly throughout childhood and into adolescence [98–103]. Age-corrected cognitive standard scores were used, and there was not a significant difference in the number of years to follow-up between the choline and placebo groups. Future choline studies could take into account age at MRI scanning and number of years since treatment. As recently illustrated in a comprehensive review, further longitudinal investigation into neurodevelopmental trajectories in PAE is needed [50]. A third limitation is the risk for inflated type 1 errors. Given the small sample size and exploratory nature of the current study, we chose to present the results without correction so as to avoid inflating the type 2 error rate (potentially missing important associations at this preliminary stage of the work) [113]. We carefully described patterns in our findings instead of focusing on isolated findings. Specifically, we found cognitive differences between the choline and placebo groups (e.g., processing speed) that were consistent across different tests, and our neuroimaging findings were consistent with previous research on white matter microstructure in children with PAE. Additional studies will be needed to replicate these findings.

## Conclusions

Results of the current 7-year follow-up study suggest continued neurodevelopmental benefits of early choline supplementation in children with FASD that are detectable into middle childhood and adolescence. The MRI results suggest that the cognitive benefits are associated with white matter microstructural effects of choline supplementation. To our knowledge, this study is the first to report on NODDI in a sample of children with FASD.

Ultimately, children with FASD are likely to benefit from a combination of supports and interventions, and our results provide further evidence that early nutritional supplementation may play a role in supporting positive long-term developmental outcomes. Results presented here highlight the importance of continuing to assess for treatment effects of early neurodevelopmental interventions that may manifest over the course of development. Ongoing efforts at the level of public health and legislation will also continue to be crucial in supporting individuals affected by FASD, including improving diagnostic capacity and early detection, supporting prenatal care and addiction treatment, and further developing behavioral and other interventions that can meaningfully benefit children with FASD across the lifespan [1, 3, 11, 114].

## Data Availability

The datasets used and/or analyzed during the current study are available from the corresponding author on reasonable request. Outcome data from the parent trial is available on ClinicalTrials.Gov.

## Abbreviations

ARND: Alcohol-related neurodevelopmental disorder
FAS: Fetal alcohol syndrome
FASD: Fetal alcohol spectrum disorders
CC: Corpus callosum
D-KEFS: Delis-Kaplan Executive Functioning System
NDI: Neurite density index
NODDI: Neurite orientation dispersion and density imaging
ODI: Orientation dispersion index
pFAS: Partial fetal alcohol syndrome
PAE: Prenatal alcohol exposure
WISC-V: Wechsler Intelligence Scale for Children, 5th Edition
WM: White matter
